# Secretory phospholipase A2 pathway in various types of lung injury in neonates and infants: a multicentre translational study

**DOI:** 10.1186/1471-2431-11-101

**Published:** 2011-11-08

**Authors:** Daniele De Luca, Ettore Capoluongo, Vincent Rigo

**Affiliations:** 1 Pediatric Intensive Care Unit, Dept of Emergency and Intensive Care, University Hospital "A.Gemelli", Catholic University of the Sacred Heart - Rome, Italy; 2 Laboratory of Clinical Molecular Biology, Dept of Molecular Medicine, University Hospital "A.Gemelli", Catholic University of the Sacred Heart - Rome, Italy; 3 Neonatal Intensive Care Unit, University of Liège, CHU de Liège (CHR Citadelle), Belgium

## Abstract

**Background:**

Secretory phospholipase A2 (sPLA2) is a group of enzymes involved in lung tissue inflammation and surfactant catabolism. sPLA2 plays a role in adults affected by acute lung injury and seems a promising therapeutic target. Preliminary data allow foreseeing the importance of such enzyme in some critical respiratory diseases in neonates and infants, as well. Our study aim is to clarify the role of sPLA2 and its modulators in the pathogenesis and clinical severity of hyaline membrane disease, infection related respiratory failure, meconium aspiration syndrome and acute respiratory distress syndrome. sPLA2 genes will also be sequenced and possible genetic involvement will be analysed.

**Methods/Design:**

Multicentre, international, translational study, including several paediatric and neonatal intensive care units and one coordinating laboratory. Babies affected by the above mentioned conditions will be enrolled: broncho-alveolar lavage fluid, serum and whole blood will be obtained at definite time-points during the disease course. Several clinical, respiratory and outcome data will be recorded. Laboratory researchers who perform the bench part of the study will be blinded to the clinical data.

**Discussion:**

This study, thanks to its multicenter design, will clarify the role(s) of sPLA2 and its pathway in these diseases: sPLA2 might be the crossroad between inflammation and surfactant dysfunction. This may represent a crucial target for new anti-inflammatory therapies but also a novel approach to protect surfactant or spare it, improving alveolar stability, lung mechanics and gas exchange.

## Background

### Phospholipase A2 biology

Phospholipases A2 are a widely distributed group of enzymes primarily implicated in the turnover of membrane phospholipids and lipid digestion. They are also crucial for the inflammation pathways, as they are the first step for the production of eicosanoids and other inflammatory mediators [1,2]. Secretory phospholipase A2 (sPLA2) is the low molecular, well conserved and secreted form of the enzyme. It is excreted into the alveoli mainly by macrophages and mast cells [1-3]. sPLA2 has a dual role, as it contributes to the inflammation pathway and it is also the main enzyme involved in the catabolism of surfactant [1,2,4] This complex proteo-lipid mixture is essential for the alveolar opening and the maintenance of an adequate gas exchange.

sPLA2 is well known to be involved in lung inflammation and surfactant degradation based on animal and human studies in adults [4]. Therefore, it is conceivable that sPLA2 through either its pro-inflammatory role or the surfactant catabolism, might be involved in the pathogenesis of several critical respiratory diseases. Basic data have shown that both sPLA2 activity and expression are regulated by many factors including steroids, Clara Cell Secretory Protein (CCSP), Tumor Necrosis Factor-α (TNFα), Surfactant protein A (SP-A) and certain surfactant phospholipids, Interleukine-1 (IL-1) and some other cytokines [4].

**Figure 1:**
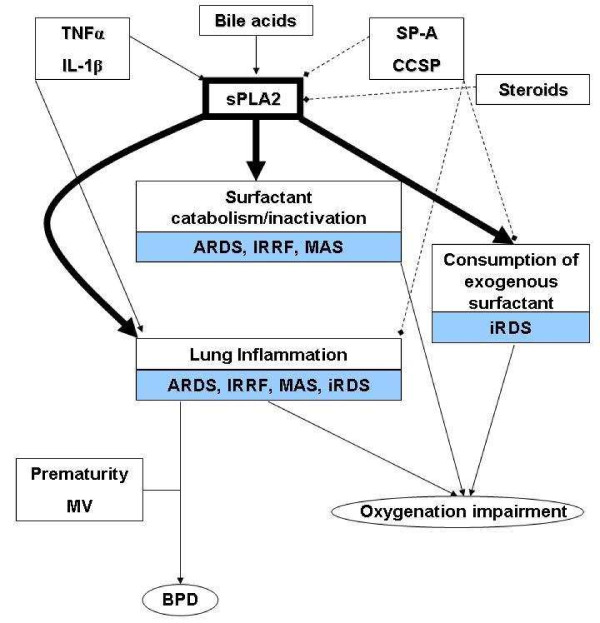
**Possible involvements of sPLA2 pathway in the pathophysiology of critical respiratory diseases in infants**. Full lines with arrows and hatched lines with squares indicate stimulatory and inhibitory actions on the enzyme activity and expression, respectively. Bold arrows show the direct consequences of the enzymatic activity in different diseases. ARDS: acute respiratory distress syndrome; BPD: broncho-pulmonary dysplasia; iRDS: infants' respiratory distress syndrome; IRRF: infection related respiratory failure; MAS: meconium aspiration syndrome; sPLA2: secretory phospholipase A2; CCSP: clara cell secretory protein; IL-1β: interleukine-1 β; SP-A: surfactant protein-A; TNFα: tumor necrosis factor-α; MV: mechanical ventilation.

Imbalance in the sPLA2 pathway due to different production of its modulators may account for increased surfactant degradation or lung tissue inflammation. Schematic representation of sPLA2 pathway is presented in Figure 1: the possible roles of the enzyme in the pathogenesis of acute respiratory distress syndrome (ARDS), infant respiratory distress syndrome (iRDS), broncho-pulmonary dysplasia (BPD), infection related respiratory failure (IRRF) and meconium aspiration syndrome (MAS) are also illustrated. Data about the sPLA2 role in each of the above-mentioned diseases are described in the following paragraphs.

### sPLA2 and ARDS

A wide body of literature suggests a role for sPLA2 in the development of ARDS and acute lung injury (ALI), its milder form. sPLA2 interferes with the surfactant activity and so reduces compliance [4-6]. sPLA2 starts a vicious cycle in which it damages the surfactant; since some surfactant components have their own inhibitory effect on sPLA2 activity and expression [7,8], the sPLA2-induced surfactant damage reduces this inhibition and thus the enzyme is able to further catabolize the surfactant phospholipids [4]. Thus, sPLA2 facilitates the action of other injurious agents against the lung epithelium, leading to further surfactant damage, alveolar collapse and respiratory impairment [4]. This process is also linked to the lung tissue inflammation, since TNFα and some other pro-inflammatory cytokines are strong sPLA2 inductors throughout the regulation of NFkB nuclear transcription factor [4,7,8]. Moreover, sPLA2 itself starts the inflammatory cascade, since it is the first step in the biochemical pathway leading to the production of arachidonic acid derivatives [9]. Inflammation may further inactivate surfactant, contributing to the above-described vicious cycle [4-6]. sPLA2 activity is raised in broncho-alveolar lavage fluid (BALF) in animal models of ARDS and in adult patients and this correlates with the clinical severity and mortality [4,5,8,10]. We recently found raised enzyme levels in post-neonatal ARDS, similarly to the adult findings [11]. Moreover, respiratory syncytial virus (RSV) infection seems to cause a more severe ARDS because of the sPLA2 over-expression, triggered by the RSV itself [12]. Consistently, transgenic animals defective for the CCSP gene, experience higher inflammation and mucous production, when infected by RSV [13].

In animal models, the administration of sPLA2 inhibitors reduced lung inflammation and improved both compliance and oxygenation, especially if the inhibitor is administered early during the injury development [8,14]. Similarly, the inhibitor was able to reduce sPLA2 activity in BALF of patients with iRDS, IRRF, MAS and post-neonatal ARDS [15].

### sPLA2 and iRDS

The importance of surfactant is well known in neonatal critical care. An inadequate surfactant production is the pivotal cause of hyaline membrane disease, also called infant respiratory distress syndrome (iRDS), the most frequent respiratory disease of preterm infants [16]. Although exogenous surfactant administration is curative in many preterm infants, long-term respiratory sequels are still a significant problem in this population, with 20% of the surviving preterm babies affected by BPD [17]. Moreover, the tiniest babies born at the limit of viability often require multiple surfactant administrations. Many of these very preterm deliveries are associated with infections and chorioamnionitis [18]. In these cases, inflammation lengthens the lung injury, decreasing the usefulness of exogenous surfactant and damaging the lung tissue [18]. In such situation, sPLA2 is likely to play a crucial role: we found increased sPLA2 levels in babies with iRDS comparing to normal term neonates [19]. sPLA2 was found to increase foetal neutrophil migration and so to enhance lung tissue inflammation [20]. Babies with higher sPLA2 activity are likely to be the ones needing repeated surfactant administrations and they are at higher risk for chronic lung disease.

Some authors recently tried to administer CCSP to preterm neonates. CCSP is a natural inhibitor of sPLA2 in the lung [21,22]. This drug has been given endotracheally together with surfactant [21] achieving a significant reduction in lung tissue inflammation. Similar results in terms of inflammatory markers and lung function have been obtained in animal models of iRDS and MAS [23-25].

Other authors have proposed the same approach with endotracheally administered budesonide, vehicled by surfactant [26]. Budesonide inhibits sPLA2 and has been associated with a decrease in the release of sPLA2-induced pro-inflammatory cytokines [27]. Surfactant is the cornerstone of iRDS therapy and the sPLA2 inhibition could theoretically protect it, reducing the need for repeated doses and improving the long-term respiratory outcome.

### sPLA2 and IRRF

During sepsis or pneumonia surfactant may be inadequately produced and recycled or it may be inactivated by lung tissue inflammation [28,29]. In these cases surfactant therapy is often less useful and does not achieve the clinical improvement usually seen in iRDS [28,29]. Mortality rate for such condition is still remarkable in term infants and even higher in preterm babies, who often experience sepsis or pneumonia as nosocomial infections acquired in the intensive care units [16]. sPLA2 is raised in BALF of neonates with IRRF [19]. This is consistent with animal and cellular studies showing that bacterial membrane lipopolysaccharide is a potent inductor of sPLA2 [4]. Nonetheless, no definite data are available about the role of sPLA2 and its pathway during IRRF.

### sPLA2 and MAS

Pancreatic sPLA2 has been indicated as a main etiological agent of MAS, one of the worst form of neonatal lung injury, characterized by massive surfactant inactivation, lung tissue inflammation and airway obstruction [30-32]. Meconium carries high amounts of sPLA2 and bile acids that are likely to contribute to lung injury, increasing sPLA2 activity and causing further surfactant inactivation [33]. Moreover, not only the pancreatic sPLA2 but also the pulmonary isoforms of the enzyme may be involved in the syndrome, [34] as lung sPLA2 production may be boosted by the meconium-induced release of pro-inflammatory cytokines [35-37] and through a specific cross-talk between different enzyme isoforms [38]. Consistently, we have recently found raised levels of pulmonary sPLA2 in BALF of patients affected by MAS when compared to their own meconium and to control babies [39]. MAS still has a mortality rate of about 50% and sometimes requires invasive treatments as broncho-alveolar lavage using saline/surfactant solutions or extra-corporeal life support [40,41].

sPLA2 is also involved in neonatal bile acids pneumonia, a more rare form of lung injury, [42] in which neonatal lungs are challenged with the bile acids coming from the maternal circulation when the mother is affected by obstetric cholestasis [43,44]. In this condition, the neonatal lung may experience a sPLA2 over-activation [33] due to the bile acids coming from maternal circulation. This may lead to severe respiratory failure, since bile acids increase the sPLA2 activity enabling the presentation of the phospholipid substrate to the catalytic site of the enzyme [45].

### sPLA2 genetics and human diseases

It is known that some sPLA2 gene polymorphisms are associated with chronic obstructive pulmonary or coronary artery disease [46,47]. Given the wide role of sPLA2 in many critical respiratory conditions, an individual predisposition due to different polymorphisms is likely to exist. Nevertheless, data about sPLA2 genetics, its association with respiratory failure and its clinical severity have not been published.

## Methods and design

### Study hypothesis and purposes

Available data allow hypothesizing a role for sPLA2 or its modulators in the pathogenesis, in the clinical severity and in the development of complications of the above mentioned types of lung injury. Our aim is to clarify such role, in order to better understand whether or not sPLA2 therapeutic inhibition might be a helpful strategy. To do that, we are planning to:

1. Identify the exact subtype(s) of sPLA2 produced and secreted into the alveoli during post-neonatal ARDS-ALI, MAS, iRDS and IRRF. This is important to know because sPLA2 inhibitors may have a different specificity for the various enzyme subtypes. In animal models, distinct sPLA2 subtypes have been associated to lung dysfunction [1,48-50].

2. Study the main modulators of sPLA2 expression and activity (TNFα, CCSP, SP-A and IL1). This will allow identification of possible pathway imbalances and eventually new therapeutic targets.

3. Clarify what happen to sPLA2 and its pathway when exogenous surfactant is administered, as it usually occurs to preterm neonates. This will allow to understand if there is a link between sPLA2 activity/overexpression, the repeated need for surfactant and BPD occurrence.

4. Clarify if there is a genetic predisposition due to different sPLA2 genes polymorphisms which could lead to more severe clinical pictures in iRDS, ARDS, MAS or IRRF or to a long term negative outcome.

### Originality of the project

This is the first study aimed at investigating the whole sPLA2 pathway in the above-described types of lung injury. To date, no study has addressed the functioning of the whole sPLA2 pathway, including the role of genetics, pathway modulators and related exogenous therapies that may affect it. This is essential because the diseases in which sPLA2 is thought to be important are basically different and the enzyme could play a different role through different subtypes, with different influence of its modulators and various response to the exogenous surfactant administration. Moreover, gene polymorphisms may play a role affecting the enzyme activity and so the clinical picture. Furthermore, many respiratory diseases potentially caused or influenced by sPLA2 are typical of newborn infants (e.g.: MAS, iRDS) or are present both in adults and in children, but with different causes and characteristics (e.g.: ARDS) [51]. Thus, data coming from animal studies or from adult experience cannot be directly applied to children and a specific study is warranted. The data coming from the present project will be crucial for future studies targeted at developing an anti-sPLA2 therapeutics.

### Project management

A multicenter design has been previewed and the project will be coordinated at the Laboratory of Clinical Molecular Biology of the University Hospital "*A.Gemelli*", Catholic University of the Sacred Heart in Rome. A Study group on Secretory Phospholipase in Paediatrics (SSPP) has been arranged and project coordinators will be a clinical pathologist/biochemist (Prof. E. Capoluongo, Head of the Lab) and a paediatric intensivist/neonatologist (Dr. D. De Luca).

SSPP will consist of two working groups for this project:

**a. Laboratory group**. This consists of biochemists and biologists experts in several molecular biology techniques applied to BALF specimens. These investigators will remain blinded to the clinical data, which will be known only to the project coordinators.

**b. Clinical group**. This will consists of all clinicians - neonatologists and/or pediatric intensivists - working in intensive care units (at the University Hospital "*A.Gemelli" *or in so called "Collaborating centers") where patients' enrolment, samples and data collection will be performed.

Clinical investigators will meet a project coordinator regularly before the beginning of the study. This will happen by tele-conference or by visiting the collaborating centre. All investigators will remain in contact during the entire project by e-mail and/or tele-conference. One of the project coordinator will also give a 24h/7d availability by phone in case of urgent matters.

The project is still open and other intensive care units are welcomed to participate as collaborating centres. Interested colleagues should contact the corresponding author to discuss the study feasibility (please see at the end of manuscript).

### Study phases

To accomplish the study purposes, the work will be subdivided in two phases:

1) clinical phase; 2) bench phase.

#### 1) clinical phase

##### Enrolment

The following group of patients will be identified: iRDS, IRRF, MAS, ARDS-ALI. To be enrolled in a group babies must fulfil all the following inclusion criteria:

A. Preterm neonates (gestational age ≤ 37 sett) with iRDS

*C-Reactive protein (CRP) < 10 mg/L or procalcitonin (PCT) < 0.6 ng/mL in the first 72 hours of life; Chest-X-rays typical for iRDS; no clinical signs of sepsis; need for mechanical ventilation*.

B. Infants and neonates with IRRF (regardless of the age)

***B1. Early IRRF. Neonates from mother with vaginal or urine positive cultures***. *Respiratory distress signs and CRP > 10 mg/L *[52]*or PCT > 0.6 ng/mL *[53]*in the first 72 hours of life; clinical signs of sepsis or blood/BALF positive culture; need for mechanical ventilation*.

***B2. Late IRRF***. *Neonates with respiratory distress signs beyond the first 72 hours of life or infants, irrespectively of the age and CRP > 10 mg/L *[52]*or PCT > 0.6 ng/mL *[53]; *clinical signs of sepsis or blood/BALF positive culture; need for mechanical ventilation*.

C. Neonates with MAS

*Neonates with meconium stained and thick amniotic fluid who required broncho-aspiration following Neonatal Resuscitation Program guidelines*[54]. *Continuous need for mechanical ventilation at 15 minutes of life. Chest-X rays typical for MAS*.

**D. Infants with ARDS-ALI **[55]

*Infants beyond neonatal age (> 30 days of life) and < 1 year of age under mechanical ventilation and having PaO_2_/FiO_2 _ratio < 200 (ARDS) or < 300 (ALI), chest-X rays typical for ARDS-ALI, acute onset of the respiratory distress and no cardiogenic oedema/increase in left atrial pressure*.

A control group has also been previewed, as follows:

E. Infants and neonates without respiratory diseases (NLD: No lung disease)

*Patients ventilated for non-pulmonary reasons (e.g.: anaesthesia, central nervous system diseases), PaO_2_/FiO_2 _ratio > 300 or FiO_2 _= 0.21, negative CRP and PCT, normal chest-X rays and chest clinical examination*.

A careful revision of the clinical characteristics will be done for each patient at the moment of discharge (or death). This will be done in each centre to ensure the appropriateness of diagnosis and internal validity.

##### Procedures to be performed in the intensive care units

**1**. Broncho-alveolar lavage.

This procedure will be performed as soon as possible from the fulfilling of the enrolment criteria. In case of neonates, broncho-alveolar lavage will be performed in the following schedule:

• PRE-SURFACTANT

• POST-SURFACTANT (after at least 12 hours from the surfactant administration)

• PRE-2^nd ^SURFACTANT (only for babies needing a second dose)

• POST-SURFACTANT (after at least 12 hours from the 2^nd ^surfactant administration)

Obviously, for infants receiving just a single surfactant dose or no surfactant at all, only one or two broncho-alveolar lavages will be carried out.

This procedure is to be intended a non-bronchoscopic lavage: it will be performed according to our previously described and well standardized technique [15] and following the advices of the European Respiratory Society guidelines [56]. All BALF specimens will be added with 0.9% saline up to 2 mL and a small aliquot of the fluids will be sent for microbiological culture.

**2**. 1.5 mL blood drawing in a vial with no anti-coagulant to be centrifuged (see below).

If a baby undergoes repeated broncho-alveolar lavages, the blood drawing will be repeated each time. Every BALF and blood specimens must be obtained within 1 hour from each other.

**3**. 0.5 mL blood drawing into an EDTA vial to be immediately stored at 4°C. This blood will be used for DNA extraction to analyse sPLA2 genes polymorphisms and will be drawn only once for each baby.

In general, blood drawings will be performed from an indwelling arterial line or from a central venous line to avoid haemolysis. Blood without anti-coagulant and BALF samples will be immediately centrifuged at 3000 rpm for 10 minutes to separate the serum or the supernatants which will be immediately stored at - 80°C.

##### Data to be registered in the intensive care units

The following data will be recorded either from the vital parameters monitors, from the ventilator screen or the clinical files.

• Name and sex

• Group of enrolment

• Type of ARDS (direct/indirect)

• Main identifiable cause of ARDS

• Pre-existing respiratory diseases (if any)

• Hours of life or hour from the fulfilment of enrolment criteria at which the samples have been obtained.

• Cumulative dose of prenatal steroids (for preterm neonates)

• Doses of steroids received till the enrolment (if any)

• Type of mechanical ventilation provided

• Peak inspiratory pressure, positive end-expiratory pressure, mean airway pressure (Pāw), Expired tidal volume (pro Kg)

• Total respiratory rate and spontaneous respiratory rate (if any)

• Dynamic compliance over ten mechanical breaths or static compliance using end-expiratory occlusion (depending on the ventilator)^§^

• Total respiratory system resistances over ten mechanical breaths

(If patients are ventilated with high frequency oscillatory ventilation, instead of the above-mentioned parameters, Pāw, amplitude and frequency will be recorded. If a specific flow-sensor [57] is available the tidal volume delivered during oscillations will also be registered and used for further calculation [see below]).

• Cumulative dose of exogenous surfactant (for neonates)

• FiO_2_

• Oxygen saturation at the right hand

• Gestational age/Birth weight or Age in months/weight

• PaCO_2 _and PaO_2_

• pH

• Base excess and Lactate

• Mean arterial pressure

• Central venous pressure (if available)

• Heart rate

• Blood urea nitrogen

These data must be recorded as close as possible to the broncho-alveolar lavage/blood drawing (max within 1 hour from such procedures). These data will be recorded in real time in an appropriate electronic database provided by the coordinating centre to each intensive care unit participating in the study.

Moreover, using the above-mentioned data, the following indexes will be calculated:

• Oxygenation index (FiO_2 _× Pāw/PaO_2_)

• PaO_2_/FiO_2 _ratio

• Ventilatory index (Peak - end expiratory pressure) × respiratory rate × PaCO_2_/1000 (if conventional ventilation is provided)

• Alveolar ventilation estimate during high frequency oscillatory ventilation (DCO_2_= frequency * (tidal volume)^2^], if a specific flow sensor is available) [58]

Moreover, the following data will be recorded:

• Mortality

• Intensive care unit length of stay

• Duration of invasive ventilation

• Duration of oxygen therapy

• Any neurological sequel at the discharge

• Oxygen requirement after discharge

• Diagnosis of chronic lung disease, for preterm neonates, according to the NICHD definition of BPD [59].

##### Exclusion criteria

Patients with one of the following characteristics will not be enrolled in any group:

1. Congenital lung malformations of any type

2. Lung or thoracic surgery

3. Lung cancer of any type

4. Congenital complex malformations

5. Patients undergoing extracorporeal life support.

##### Storage and transfer of data and samples

All data will be anonymously stored in the above described electronic database and will remain property of the enrolling centre. At the end of the clinical phase they will be checked for validity in each centres and then sent in a secured way to the Coordinating centre. At that time all specimens will be also sent under dry ice to the Coordinating centre.

#### 2) Bench phase

##### sPLA2 pathway

In serum and BALF supernatants the following analyses will be performed:

• Western blotting for sPLA2-IIA, -V, -X. For this procedure external (actinin) and internal (recombinant human sPLA2 subtypes, -IIA, -V, -X) controls will be used and total protein measurements will also be performed with Bradford's method [5].

• TNFα assay

• SP-A assay

• IL1 assay

These assays will be performed using specific ELISA/EIA kits already used to analyse BALF. These methods have been proven to do not cross-react with other cytokines and with sPLA2; in previous studies, coefficients of variation of the standard curve resulted always ≤ 9% [15,39,60-62].

• sPLA2 global activity assay

To do this assay, all samples will be centrifuged (for 10' at 12000 rpm and then for 3' at 3500 rpm) through a membrane-filter with a molecular weight cut-off of 30 kDa (Amicon Ultra centrifugal filter; Millipore, Billera, MA-USA), to separate the secretory and cytosolic phospholipases (which weight ≈14 kDa and ≈80 kDa, respectively) [39,62,63].

• High sensitivity urea nitrogen assay in the BALF supernatant.

All measurements in BALF will be corrected for the serum-to-BALF urea ratio, as previously described [64].

##### sPLA2 genetics

sPLA2-IIA [HGNC:9031], -V [HGNC:9038] and -X [HGNC:9029] genes polymorphisms will be studied in the patients' leukocytes. We found 15 single nucleotide polymorphisms (SNP) for these genes. These polymorphisms were searched in the dbSNP (http://www.ncbi.nlm.nih.gov/SNP/), JSNP (http://snp.ims.u-tokyo.ac.jp), GenBank at the NCBI (http://www.genbank.com) and Applied Biosystems genotyping databases (http://www.appliedbiosystems.com), as well as in a previous study linking them to coronary artery disease [47]. Analysis of genetic polymorphisms SNP genotyping will be performed by TaqMan allelic discrimination assay [65]. Polymerase chain reaction will be performed with specific primers at concentrations of 900 nM. Fluorescence data files from each plate were analyzed by a specific software. In order to verify the correct genotype assignment, we will randomly analyse some of the above screened samples by means of genetic sequencing (BigDye terminator technique).

All laboratory procedures will be carried out respecting safety regulations and bench investigators will be blinded to the patients' group of origin and to their clinical data. To accomplish this blindness, before starting the bench phase all vials will be re-labelled with a new code and only the project coordinators will be aware of the new code.

##### Statistics and sample size

Data will be tested for normality and then analyzed with parametric or non-parametric procedures, as appropriate. Accordingly, univariate analysis using Student and analysis of variance or Mann-Whitney, Wilcoxon, Kruskal-Wallis and Friedman tests will be performed. Some laboratory data will be subjected to correlation analysis with clinical findings, using Pearson's, Spearman's or Kendall's technique, according to data characteristics.

Subsequently, if needed, significant results will be subjected to multiple curve estimation procedure [66] and/or multivariate analysis according to data characteristics and the results of the univariate analyses. The genetic data will be analyzed using χ^2 ^test for the Hardy-Weinberg equilibrium of alleles at the individual loci. The association between genotypes and clinical data will be tested with χ^2^- or Fisher test and then with logistic regression or analysis of co-variance, as appropriate [67]. All statistical analyses will be performed by the project coordinators, who have a long experience and formal training in biostatistics.

Despite a formal sample size calculation is not warranted, based on the available data [5,11,19], we previewed a convenience sample size, as follows: 50-60 preterm infants affected by iRDS, with at least 20 receiving a second surfactant dose; for ARDS and IRRF groups 20 patients will be also convenient, while 10 control neonates or infants will be considered. These sample size have been checked for power regarding the correlation between sPLA2 activity and two selected clinical variables (using Power and Precision demo rel. 3.2 [68]). Given α-error of 0.05 and a correlation coefficient (*r*) ≥ 0.6 and ≥ 0.85 [5,11,19] for the respiratory compliance and the PaO_2_/FiO_2 _ratio, respectively, the power resulted > 80% in both cases.

The study is supposed to last 12-18 months for the enrolment phase in each collaborating centre, and 3-6 months for the laboratory phase at the coordinating centre.

## Ethical considerations

The protocol and consent form have been approved by the Ethical Committee of the University Hospital "*A.Gemelli*" at the Catholic University of the Sacred Heart (Rome, Italy) as coordinating centre. Local ethical boards in each collaborating centre have also approved the protocol.

The participation to the study will not change in any way the routine clinical assistance previewed for every patient. Furthermore, the participation to the study will respect all the local Regulations about safety procedures and the privacy. Informed consent will be given by parents or tutors of each baby, before the enrolment in the study.

## Publication policy

Study results will be presented to each investigator by teleconference and/or e-mail. If possible a meeting in occasion of one of the major congresses in the field of Paediatrics or Critical Care (like the European Society for Paediatric Research or European Society for Paediatric and Neonatal Intensive Care Congresses) will be organised. Data will be also presented at these meetings and the subsequent manuscripts will be circulated between all investigators for revision. All resulting manuscripts will be authored by the project coordinators and by the group authorship (SSPP: Study group on Secretory Phospholipase in Paediatrics).

## Discussion

This study, thanks to its multicenter design, will clarify the role(s) of sPLA2 and its pathway modulators in several paediatric and neonatal forms of lung injury. In fact, enough evidence is available to indicate sPLA2, or at least some of its subtypes, as a key point in the pathophysiology of certain critical respiratory diseases. Inflammation is a complex process and is essential in many of these conditions: sPLA2 might be the main crossroad between inflammation and surfactant catabolism. Since this latter is surely an important component of some clinical situations, sPLA2 is worth to be studied in its metabolic and genetic issues, trying to correlate them to the clinical pictures.

Given the peculiarity of such diseases and the relative rarity of some of them, only a multicentre design will be able to clarify this field. The purpose is not free from practical consequences, as several sPLA2 inhibitors are now available or under advanced development [69]. Many drugs are already used in respiratory critical care [70] but sPLA2 blockade might represent a new anti-inflammatory therapy and a novel approach to protect surfactant or spare it, improving alveolar stability, lung mechanics and gas exchange.

The establishment of a net of centres involved in clinical research, along with the support of bench investigators, will help in understanding this field and in building future randomised interventional studies.

## List of abbreviations

sPLA2: secretory phospholipase A2; CCSP: Clara cell secretory protein; SP-A: Surfactant protein A; ARDS: acute respiratory distress syndrome; ALI: acute lung injury; BALF: broncho-alveolar lavage fluids; iRDS: infant respiratory distress syndrome or hyaline membrane disease; BPD: broncho-pulmonary dysplasia; MAS: meconium aspiration syndrome; IRRF: infection related respiratory failure; RSV: respiratory sincytial virus; SSPP: Study group on Secretory Phospholipase in Pediatrics; CRP: C-Reactive protein; PCT: procalcitonin; Pāw: mean airway pressure; DCO_2_: Alveolar ventilation estimation during high frequency oscillatory ventilation; cPLA2: cytosolic phospholipase; SNP: single nucleotide polymorphisms.

## Competing interests

The authors declare that they have no competing interest.

## Authors' contributions

DDL conceived the study design, created the multicentre network and visited the collaborating centres; he prepared the clinical phase, previewed the statistical analysis and drafted the manuscript. VR supervised the study design, helped in the creation of the multicentre net and drafted the manuscript. EC supervised the study design, identified the laboratory technique and the bench working program and drafted the manuscript. All members of the SSPP, contributed to the design of the study, discussed its practicability and provided what was needed to start the study in each centre, they all revised and approved the project and the final manuscript.

## Footnote

^§ ^Compliance measurement depends on the type of ventilator. Those with the end-expiratory occlusion will provide a static measure, otherwise a dynamic measurement will be done using the hot-wire anemometer flow sensor. In that case, to increase the measurement accuracy, the spontaneous breathing must be temporarily avoided, gas leaks must be < 5% and stable respiratory conditions with minimal airway secretions must be achieved. Conditions and technique for this measurement have been described in details elsewhere [15].

## Pre-publication history

The pre-publication history for this paper can be accessed here:

http://www.biomedcentral.com/1471-2431/11/101/prepub
